# Effects of Molar Ratio and pH on the Condensed Structures of Melamine-Formaldehyde Polymers

**DOI:** 10.3390/ma11122571

**Published:** 2018-12-17

**Authors:** Taohong Li, Ming Cao, Bengang Zhang, Long Yang, Guanben Du

**Affiliations:** 1 The Yunnan Province Key Lab of Wood Adhesives and Glued Products, Southwest Forestry University, Kunming 650224,China;caoming_happy@126.com (M.C.); zbg18082968142@163.com (B.Z); long133109070@126.com (L.Y.); 2 Key Lab for Forest Resources Conservation and Utilisation in the Southwest Mountains of China, Ministry of Education, Southwest Forestry University, Kunming 650224, China

**Keywords:** molar ratio, pH, methylene bridges, ether bridges, quantitative ^13^C-NMR

## Abstract

The base-catalyzed melamine-formaldehyde (MF) reactions were studied in both diluted and concentrated solutions. The influences of F/M molar ratio and pH on the polymer structures were investigated based on the quantitative ^13^C-NMR analysis. The results show that both F/M molar ratio and pH influence the competitive formation of ether and methylene bridges. For the cases of F/M = 2.0, and 3.0, methylene bridge formation is minor in contrast to ether bridges either at pH = 9.3–9.8 or at 7.3–7.8. When the molar ratio was lowered to 1.0, methylene bridges became competitive with ether bridges at pH = 9.3–9.8, but the latter is still more favorable. When the lower molar ratio overlaps with the lower pH, significant changes were found. The content of methlylene bridges was over three times that of ether bridges with M/F = 1.0 and at pH = 7.3–7.8. The results in this study were compared with those previously obtained for base-catalyzed urea-formaldehyde reactions. It was found that molar ratio and pH influence the structures of the MF and UF polymers in similar ways. The different synthesis conditions of UF and MF resin were also addressed by comparing the structures of UF polymers with MF polymers.

## 1. Introduction

Reactions of melamine with formaldehyde under certain conditions (pH, molar ratio, and temperature) produce melamine-formaldehyde polymers, namely MF resins, which are widely used as molding compounds, surface coating materials, adhesives, etc. [1]. This article focused on the synthetic conditions of MF resins used as wood adhesives. When MF resin is used as wood adhesive it exhibits good bonding strength, high stability toward hydrolysis, and low formaldehyde emission [1,2]. However, its applications were limited by its defects, especially high manufacturing cost and poor storage stability. In contrast, urea-formaldehyde resin (UF) which is currently the most widely used wood adhesive, has advantages of much lower cost and better storage stability [2,3]. However, UF resin has poor water resistance and a higher level of formaldehyde emission.

Regardless of the different performances of MF and UF, an interesting issue is their different synthesis conditions. Many studies have found that [3,4,5,6,7,8,9], under alkaline condition, addition reactions between urea and formaldehyde that produce hydroxymethylureas is the dominant process and minor condensations can occur, but only produce oligomers containing ether bridges (–NR–CH_2_–O–CH_2_–NR–). Condensations among the hydroxymethylureas that generate larger UF polymers with methylene bridges (–NR–CH_2_–NR–) require acidic conditions (generally pH = 4.0–5.5). Differently, MF resin used as wood adhesive is generally synthesized under alkaline conditions (pH = 8.0–10.0). Does this mean that base-catalyzed MF reactions are faster than UF reactions and mainly produce methylene bridges? To balance the performances and cost, scientists have been trying to synthesize co-condensed melamine-urea-formaldehyde resin (MUF) during the past decades, especially some studies reported the synthesis of MUF resins under alkaline conditions [10,11,12,13]. However, formation of real MUF co-condensed polymer structure should be doubted since UF and MF require different synthesis conditions [14].

Theoretically, urea and melamine are similar in chemical structure. Particularly, they have amino groups bonded to sp^2^ carbon atoms and p-π conjugation effects can be applied to both of them. They may have different reactivity, but the reaction types should be similar. How do we explain their different synthesis conditions? Our recent detailed study on alkaline UF reactions showed that 20–30% formaldehyde was converted to ether bridge and there was almost no methylene bridge formed under conditions of F/U = 2.0 and pH = 9.0 [15]. However when the F/U ratio was lowered to 1.0, the methylene bridges appeared to be competitive with ether bridges. This indicated that the molar ratio is also an important factor that influences the competitive formation of the two condensed structures. However, for MF reactions, the general knowledge in earlier literatures only described and emphasized the influence of pH [1,2]. Specifically, it was believed that ether bridges are favored at pH above 9.0 while methylene bridges are dominant at pH = 7.0–8.0. This is confusing because pH drops from 9.0 to 8.0 do not change the alkaline reaction media. Why were the reaction results significantly changed by a slight change of pH? This very fundamental issue has not been well addressed so far. According to the results obtained for UF reactions, the influence of the F/M molar ratio should not be ignored.

MF polymer structures were investigated by in some earlier NMR studies [16,17,18] and these studies pointed out that both methylene and ether bridges can be formed in MF reactions. However, quantitative results have not been provided to address how pH and molar ratio influence the polymer structures. Therefore, in this study, MF reactions under different pHs and molar ratios were re-examined based on quantitative ^13^C-NMR analysis of the reaction products.

## 2. Experiments

### 2.1. Preparation of MF Samples

The analytical reagent grade (AR) melamine, sodium hydroxide (NaOH), and formaldehyde (37%, wt %) containing about 7% methanol were bought from Sinopharm Chemical Reagent Co., Ltd., Shanghai, China.

In typical MF synthesis, the formaldehyde to melamine ratio (F/M) is generally around or above 2.0. Under this circumstance, melamine can dissolve in short time. However, in this work, the situation of F/M = 1.0 was considered and melamine cannot dissolve completely. Therefore, we firstly investigated the MF reaction in diluted solution and controlled the melamine concentration to be 1.0 mol/L. Two pH values, 9.3–9.8 and 7.3–7.8, were considered. For each pH, the F/M molar ratios of 3.0, 2.0, and 1.0 were investigated. To compare the reaction results in diluted solution with the results in concentrated solution, normal MF resins were also synthesized by directly mixing melamine with 37% (wt %) formalin solution. The specific procedures were described below:

Group A samples: The formalin solution was charged into a stirred reactor, the pH was brought to 9.8 using 20% (wt %) NaOH solution. Then melamine and distilled water were added to the reactor to obtain the melamine concentration of 1.0 mol/L. The pH was readjusted to be 9.8 and the solution was heated to 70 °C. After the melamine was dissolved completely, the temperature was increased to 90 °C and maintained for 90 min. During the reaction time, the pH was checked every 5 min and maintained at 9.3–9.8. The samples taken for M/F = 3.0, 2.0 and 1.0 were marked as A1, A2, and A3, respectively. Using the similar procedure, 37% formalin and melamine were mixed directly without dilution to synthesize an MF resin. The F/M ratio was controlled to be 2.0. The reaction was terminated when the cloudy dispersion of liquid resin in cold water (cloud point) was observed. This sample was marked as A4.

Group B samples: Using the above procedures, the MF reactions were investigated at pH = 7.3–7.8. The samples for MF reaction products in diluted solution were marked as B1, B2, and B3 for F/M = 3.0, 2.0, and 1.0, respectively. The sample for normal MF resin with F/M = 2.0 was denoted as B4.

### 2.2. ^13^C-NMR spectroscopy

The ^13^C nuclear magnetic resonance (^13^CNMR) spectra were measured using a Bruker AVANCE 600 spectrometer (Bruker Corporation, Billerica, MA, USA).

For each sample, a 400 μL liquid MF sample was directly mixed with 100 μL of dimethyl sulfoxide (DMSO-*d*_6_) for ^13^C-NMR determination. The spectra were recorded with a pulse angle of 90° (12 μs). A 6 s relaxation delay was used to secure quantitative results of methylenic carbons, which had *T*_1_ values of 0.16 s or smaller, measured by the inversion recovery method [14]. To achieve a sufficient signal-to-noise ratio, the inverse-gated proton decoupling method was used. The spectra were recordedat 150 MHz with 400–600 scans accumulated. The observed chemical shifts were assigned by referring to the assignments in the literature [14,16,18].

Except for the methanol peaks at 50 ppm and methoxyl ether (–OCH_3_) at 56 ppm, the peaks that were smaller than 100 ppm from methylene carbons were integrated and summed. The relative contents (%) (or molar distribution) of all methylene carbons were calculated into percentages as the ratio of the integral value of each type of methylene carbon over the total value of all methylene carbons. The relative contents of melamine ring carbon atoms were not calculated due to the serious overlap.

## 3. Results and Discussion

Although classic theory for UF resin synthesis pointed out that pH is the key factor that determines what reactions can occur, our experiments and theoretical calculations have clarified that the F/U molar ratio also plays an important role that influences the competitive formations of different condensed structures [15,19]. The calculated kinetic energy barriers for base-catalyzed UF condensation reactions indicate that formation of ether bridges is more favorable than methylene bridges, but the effects of the F/U ratio should not be ignored. A higher F/U molar ratio results in a higher substitution degree of urea and also means a higher concentration of the hydroxymethyl group. Thus, statistically, collisions between hydroxymethyl groups to form ether bridges have a higher probability than collisions between hydroxymethyl groups with amino groups. On the other hand, substitution of amino groups causes steric hindrance that suppresses the formation of methylene bridges. However, when the F/U molar ratio was lowered to 1.0, methylene bridges became much more competitive because free amino groups have a higher concentration under such conditions and reactions between free amino groups and hydroxymethyl groups are not affected by steric hindrance. Even under acidic conditions, the effect of the molar ratio appeared to be significant [19]. This effect can also be seen in this study for MF reactions.

**Figure 1:**
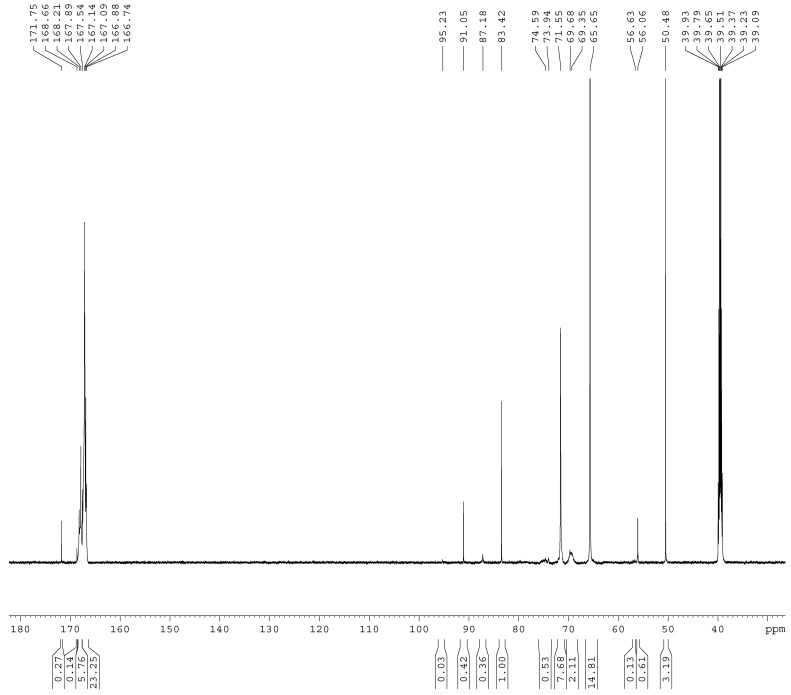
The ^13^C NMR spectrum of sample A1.

**Figure 2:**
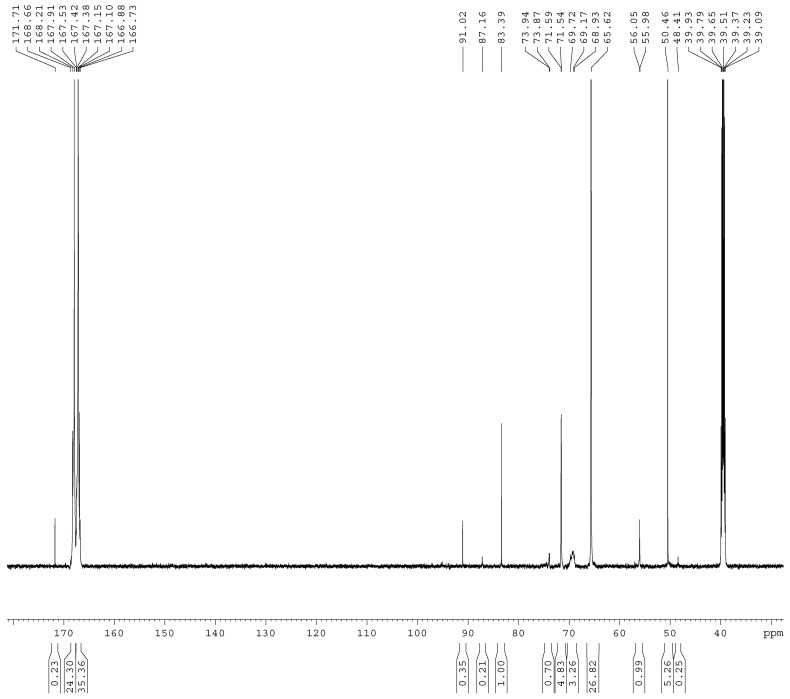
The ^13^C NMR spectrum of sample A2.

**Figure 3:**
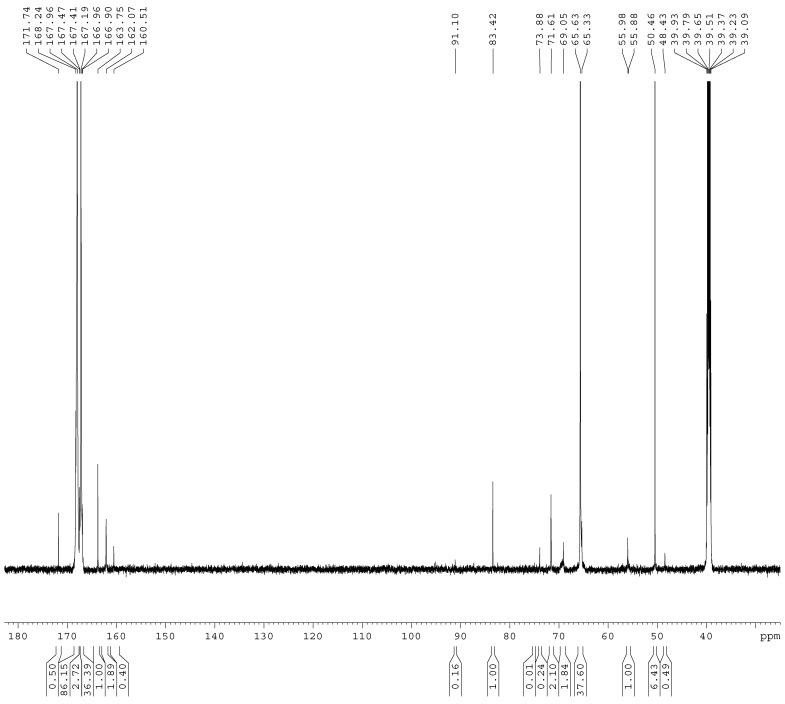
The ^13^C NMR spectrum of sample A3.

**Figure 4:**
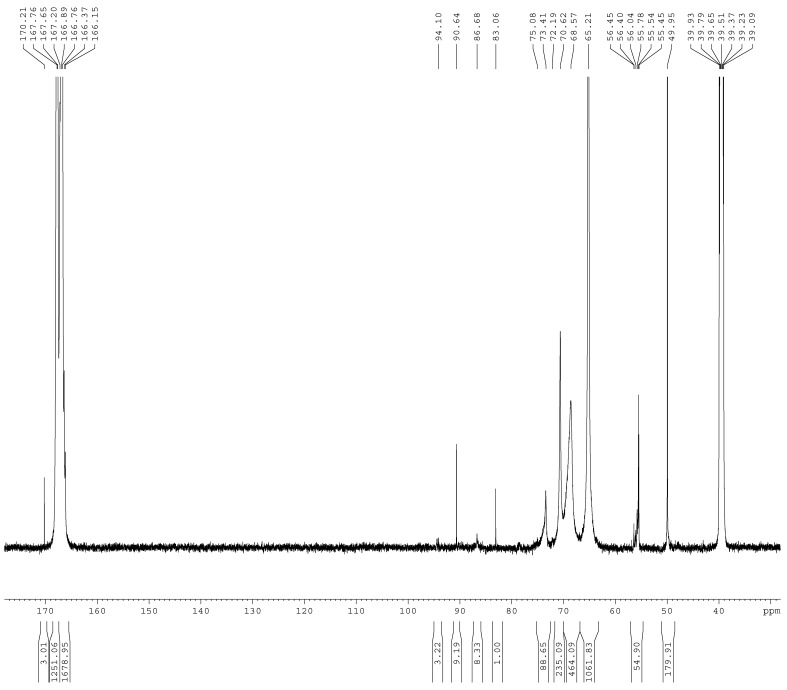
The ^13^C NMR spectrum of sample A4.

The ^13^C-NMR spectra of the samples A1–A4 are shown in Figure 1, Figure 2, Figure 3 and Figure 4. The relative contents of the methylene carbons were calculated in percentage and are listed in Table 1. To make it is easy to understand the competitive formation of the two structures, the ratio of methylene bridges over ether bridges (M/E) was calculated by considering that one methylene bridge contains one carbon while one ether bridge contains two. When the pH was controlled to be 9.3–9.8 with F/M = 3.0, all three types of methylene bridges at 48–49, 53–55, and 60–61 ppm were not observed, whereas the linear ether bridge at 68–70 ppm was almost exclusively formed. This result is similar to the case of UF under conditions of F/U = 2.0, pH = 9.0 [15].

As it can be seen in Figure 2, for sample A2, a small but obvious peak at 48.41 ppm that corresponds to a type I methylene bridge appeared when the F/M ratio was lowered to 2.0. The data in Table 1 show that the ether bridge carbon at 68–70 was also dominant (8.71%) but the methylene bridge has become a competitive process, although it was still minor (0.67%). When the molar ratio was further lowered to 1.0 (A3), the methylene carbon contents of methylene and ether bridges were measured as 1.13% and 4.24%, respectively. The ratio of methylene over ether bridge (M/E) was calculated to be 0.53, indicating methylene bridge formation became much more competitive in comparison with A1 and A2. This is also similar to the case of UF with F/U = 1.0 [15]. Thus, for MF reactions, the F/M molar ratio is also an important factor that influences the distribution of different condensed structures. 

To further confirm the distribution of methylene and ether bridge in a normal MF resin, we synthesized the MF resin (sample A4) with F/M = 2.0 at pH = 9.3–9.8. The reaction was terminated when the cloud point was observed at about 65min. Interestingly, methylene bridge disappeared and ether bridge was exclusively formed. A2 and A4 were obtained with the same molar ratio and pH, but how do we understand the observation of the methylene bridges in A2? In our recent study [20], the UF reactions were accelerated at higher pH (>12.0). The formation of the methylene bridges was not observed at the first hour. However, with the undergoing reaction, methylene bridges appeared and accompanied with conversion of a portion of ether bridges into methylene bridges, and at the late stage the content of methylene bridges exceeded that of ether bridges. This clearly indicated that competitive formations of methylene and ether bridges are controlled by their kinetic and thermodynamic nature. At pH above 9.0, formation of ether bridges is kinetically faster, but methylene bridges are thermodynamically more stable. That is to say, formation of methylene bridges takes a longer time. Thus, we can understand the different results of A2 and A4 since the reaction time for A2 (90 min) was longer than the time for A4 (65 min). Minor methylene bridges in A4 may also be observed if the reaction was allowed to continue for a longer time. These discussions remind us that the end point represented by a certain physical phenomenon (like cloud point) in normal resin synthesis may be far from the equilibrium state of the reaction system. Therefore, the resin structures formed when the reactions were terminated would be mainly determined by the kinetics of the involved competitive reactions. 

**Figure 5:**
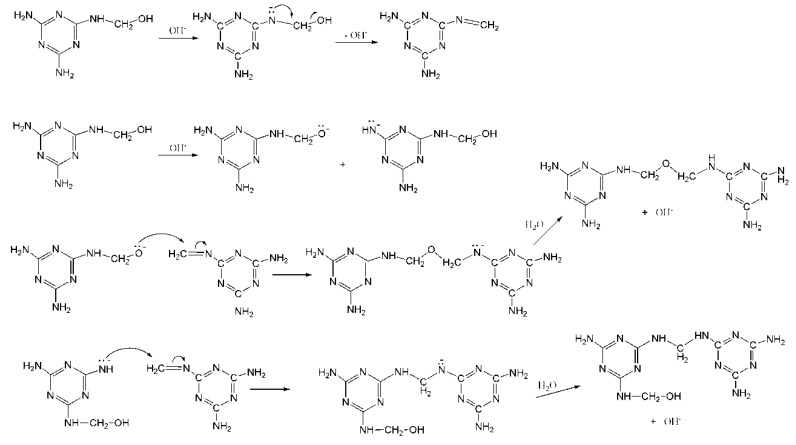
The proposed mechanism for base-catalyzed melamine-formaldehyde condensation reactions.

Since the observed MF reactions results are basically similar to those we observed for UF reactions at alkaline pH [15], here we proposed a similar mechanism for MF condensations, as shown in Figure 5, according to the reaction mechanism we recently revealed for base-catalyzed UF condensations [21]. In such a mechanism, once the Schiff’s base intermediate is formed, methylene ether and methylene bridges can be formed but the former is faster. The Schiff’s base intermediate was also proposed in an earlier study and the authors believed such an intermediate was produced by scission of ether bridges [22]. However, this mechanism cannot explain the formation of ether bridges itself. Therefore, we prefer that such an intermediate can originate from hydroxymethylmelamine.

**Figure 6:**
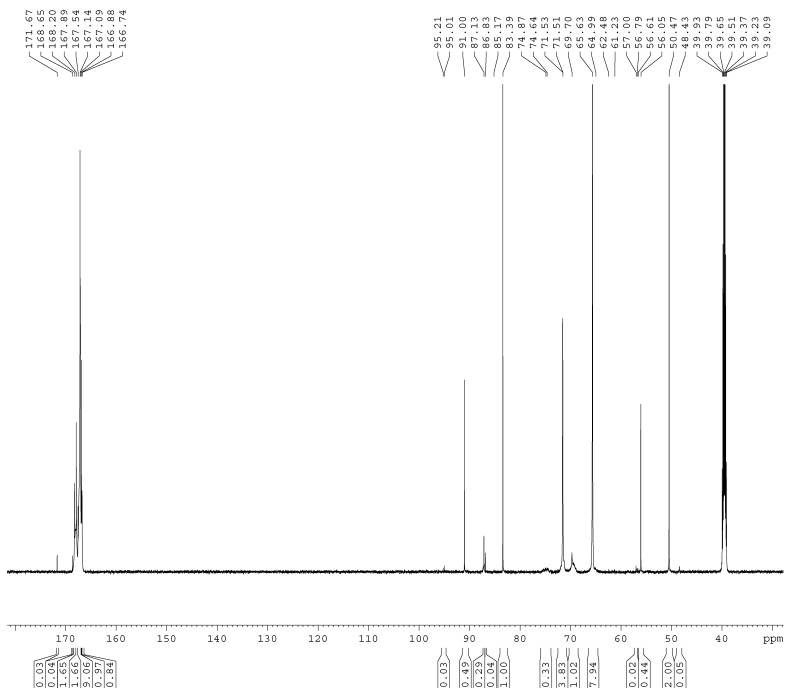
The ^13^C NMR spectrum of sample B1.

**Figure 7:**
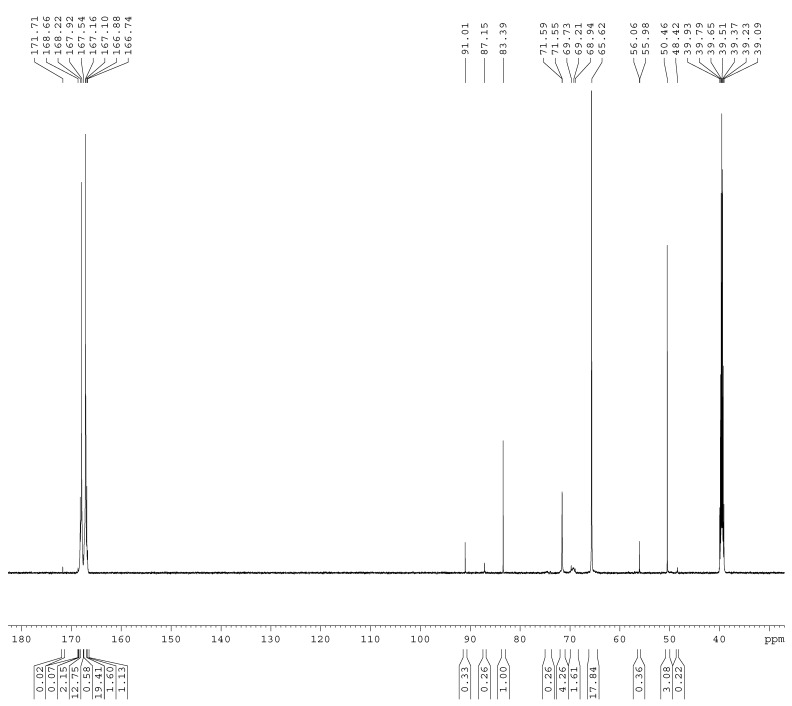
The ^13^C NMR spectrum of sample B2.

**Figure 8:**
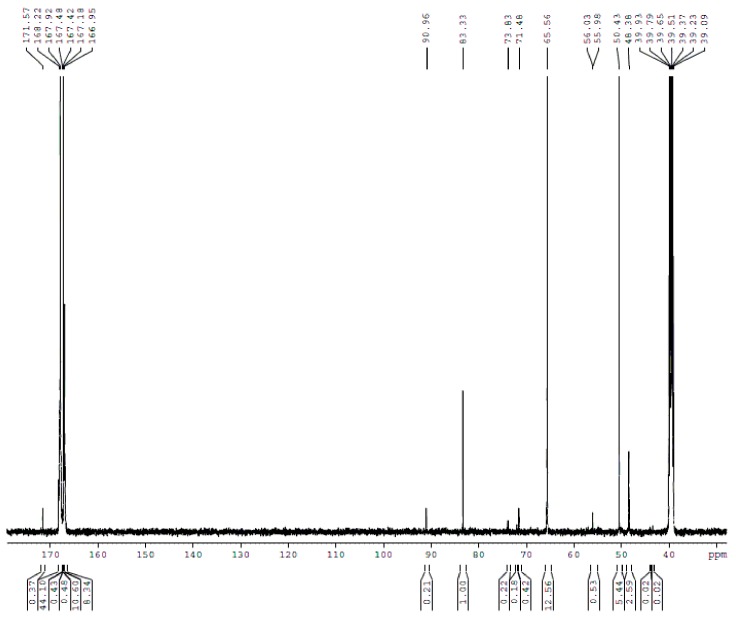
The ^13^C NMR spectrum of sample B3.

**Figure 9:**
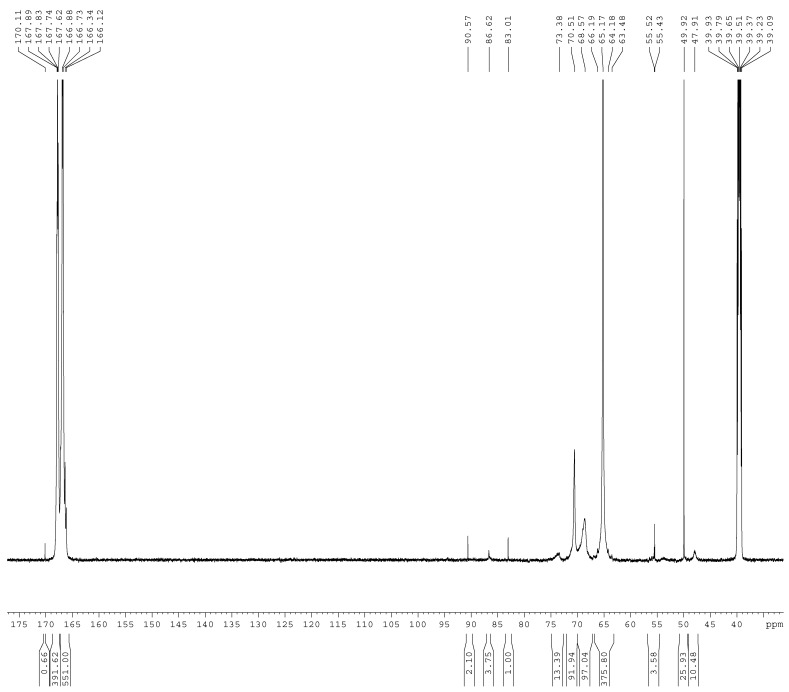
The ^13^C NMR spectrum of sample B4.

To investigate the MF reactions at lower pH, we carried out the reactions at pH = 7.3–7.8, also with F/M = 3.0, 2.0 and 1.0. The ^13^C-NMR spectra for samples B1–B4 are shown in Figure 6, Figure 7, Figure 8 and Figure 9 and the quantitative results are also listed in Table 1. Interestingly, the methylene bridge appeared in sample B1 with F/M = 3.0. Although it is still minor (0.33%) compared with ether bridges (6.79%), this result shows that the pH does influence the competitive relationships of the two condensed structures because methylene bridges were absent in A1. When the F/M was lowered to 2.0 in sample B2, the methylene bridges became more competitive as the content increased to 0.85%. Surprisingly, when the F/M ratio was further lowered to 1.0 in sample B3, methylene bridges became dominant as the methylene/ether ratio was calculated to be 3.91. How do we explain such a significant change? An important factor may be the Cannizzaro reaction. At very weak alkaline pH (7.3–7.8), the Cannizzaro reaction may be minor, but we did observe a drop of pH. Although the pH was frequently adjusted, the produced formic acid that was not neutralized immediately under weak alkaline conditions due to its weak acid nature may catalyze the reactions. Even though the formic acid did not directly catalyze the reactions, it changed the concentration of OH^−^ and H^+^ ions in solution. As MF reactions can be catalyzed by both base and acid, the role of OH^−^ and H^+^ at different pHs must be considered. When the concentration of hydroxide ion (OH^−^) remains at a relatively higher level (for example pH > 9.0), the reactions appeared to be base-catalyzed. Once the concentration of OH^−^ became lower, the concentration of proton (H^+^) became higher although the pH was still slightly above 7.0, the reactions exhibited acid-catalyzed features. Fast formation of methylene bridges here is very similar to the case we recently observed for UF reactions with F/U = 1.0, even under very weak acidic conditions (pH = 6.0) [19]. Theoretical calculations revealed that proton-catalyzed reactions between free amino groups (–NH_2_) and hydroxymethyl groups is the fastest condensation reaction [19]. The fact that the MF reaction is very fast and difficult to control when the pH is lower than 6.0 indicates that the condensation reactions are very sensitive to proton concentration. Therefore, at a pH around 7.0, the MF reaction may be catalyzed by both OH^−^ and H^+^. However, the fact that the methylene bridges were minor in samples B1 and B2 suggested that a higher F/M molar ratio still suppressed the formation of methylene bridges even at lower pH. As a result, fast formation of methylene bridges occurred only when the lower pH overlapped with the lower molar ratio.

In sample B4, which is a synthesized MF resin using 37% formaldehyde with F/M = 2.0, the M/E ratio is 0.22, which is close to that of B2 obtained in diluted solution. Based on the quantitative results of A4 and B4, it should be safe to conclude that MF polymers synthesized under conditions of F/M ≥ 2.0 and pH > 7.0 contains mainly ether bridges. The results of this study suggest that both molar ratio and pH should be claimed when the competitive formations of methylene and ether bridges are discussed.

**Figure 10:**
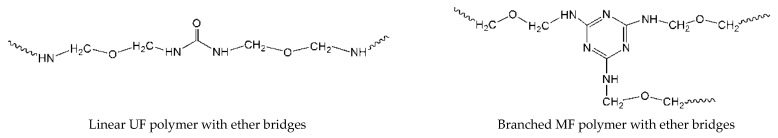
The UF and MF polymer structures containing ether bridges.

By summarizing the above discussions, we can conclude that the MF and UF reactions under alkaline conditions are basically similar. However, how do we explain the fact that the MF resin can be synthesized under alkaline conditions but synthesis UF resin requires acidic conditions? Generally, an MF resin with normal bonding strength can be obtained within 1–2 h at pH ≈ 9.0. However, according to our experience, a usable UF resin cannot be obtained at pH ≈ 9.0 even when the reaction time is longer than 24 h. Are MF reactions much faster than UF reactions? By surveying the data in the literature, we found for UF reactions that about 20–30% formaldehyde can be converted to condensed methylene ether carbons under alkaline conditions within 1h. A similar situation was found for MF, as it can be seen in this work for samples A4 and B4. Therefore, MF reactions under alkaline conditions are not faster than UF reactions. As for the condensed structures of the polymers, we have seen that the melamine molecules are also mainly linked by ether bridges when F/M is above 2.0. By comparing the UF polymer structure obtained under alkaline condition with that obtained under acidic conditions, we found the branching degree of the polymers is the key difference. In UF resin polymers that were formed in acid-catalyzed reactions, about 15–30% (depending on F/U molar ratio) formaldehyde can be converted to branching methylene carbons in the structures of –NH–CH_2_–N(–CH_2_–)– and (–N(–CH_2_)–CH_2_–N(CH_2_–)). These structures undergo further condensations during the cure process and form a cross-linking network. However, as it is shown in Figure 10, UF polymers formed under alkaline conditions mainly contain linear ether structures that cannot cure. Differently, melamine has three amino groups in the *meta-* position in a triazine ring. Such a skeleton makes the polymers bear a branched structure even though the condensed ether bridges themselves are linear. It was expected that branched ether bridges (–NH–CH_2_–O–CH_2_–N(–CH_2_–) or (–NH(–CH_2_)–CH_2_–O–CH_2_–N(–CH_2_–) may be formed in UF reactions if higher F/U molar was provided, however, they were found to be very minor. Instead, intramolecular condensations of hydroxymethylureas occurred, leading to a cyclic ether structure, namely uron species with F/U ≥ 2.0 [15,20].Therefore, the basic structural requirement for a thermosetting resin is the branching degree of the polymers, not the type of the condensed structures (ether or methylene bridges). This theory can explain the fact that a UF resin synthesized under acidic conditions, but with low F/U molar ratio (F/U ≤ 1.0), mainly contains linear structures and exhibit poor bonding strength.

It was widely accepted that the better water resistance of MF resin than UF resin can be attributed to the higher stability of methylene bridges in MF polymers toward hydrolytic attack. However, this theory should be reconsidered since MF polymers also mainly contain ether bridges. The alkalinity of the three sp^2^ hybridized nitrogen atoms in the aromatic ring of the melamine molecule may be the main reason. These nitrogen atoms can act as a buffer that capture protons and slow down the drop of pH. In other words, these nitrogen atoms have stronger proton affinity than those nitrogen atoms in condensed bridges. As a result, the hydrolysis of the condensed structure can be largely avoided or delayed. 

## 4. Conclusions

^13^C-NMR quantitative characterizations of MF polymers that were synthesized under alkaline conditions indicated that both F/M molar ratio and pH influence the competitive formation of ether and methylene bridges. For the cases of F/M = 2.0, and 3.0, methylene bridge formation is minor in contrast to ether bridges either at pH = 9.3–9.8 or at 7.3–7.8. When the molar ratio was lowered to 1.0, methylene bridges became competitive with ether bridges at pH = 9.3–9.8 but the latter is still more favorable. When the lower molar ratio overlaps with the lower pH, significant changes were found. The content of methlylene bridges were over three times that of ether bridges with M/F = 1.0 and at pH = 7.3–7.8

The MF reaction results of this study were compared with those observed previously for UF reactions under alkaline condition. It was found that the molar ratio and pH influences the two systems in similar way. But the difference in structure between melamine and urea makes different structures of MF and UF polymers, namely MF polymers have a branched structure that guarantees their curing behavior and bonding strength, whereas UF polymers containing mainly ether bridges are basically linear and cannot behave like an adhesive.

## Figures and Tables

**Table 1:** The relative content of the methylenic carbons (%).

Structures	Chemical shift (δ)	PH = 9.3–9.8				PH = 7.3–7.8			
		A1 F/M = 3/1	A2 F/M = 2/1	A3 F/M = 1/1	A4 F/M = 2/1	B1 F/M = 3/1	B2 F/M = 2/1	B3 F/M = 1/1	B4 F/M = 2/1
–NH–CH_2_–NH– (I)	48–49	-	0.67	1.13	-	0.33	0.85	10.68	1.76
–NH–CH_2_–N= (II)	53–55	-	-	-	-	-	-	-	-
=N–CH_2_–N= (III)	60–61	-	-	-	-	-	-	-	-
	Total	-	0.67	1.13	-	0.33	0.85	10.68	1.76
–NH–CH_2_OCH_2_NH– (I)	68–70	7.83	8.71	4.24	24.80	6.79	6.25	5.46	16.30
–NH–CH_2_OCH_2_N= (II)	75–77	-	-		-	-	-	-	-
=N–CH_2_OCH_2_N= (III)	78–80	–	-	-	-	-	-	-	-
	Total	7.83	8.71	4.24	24.80	6.79	6.25	5.46	16.30
M/E		-	0.15	0.53	-	0.10	0.27	3.91	0.22
–NH–CH_2_OH (I)	64–66	54.97	71.67	86.56	56.74	52.86	69.20	74.38	63.11
–NH(–CH_2_)–CH_2_OH (II)	71–72	28.51	12.91	4.83	12.56	25.50	16.52	1.91	15.44
	Total	83.48	84.58	91.39	69.30	78.36	85.72	76.29	78.55
HO–CH_2_–OH	83–84	3.71	2.67	2.30	0.05	6.66	3.88	2.73	0.17
HOCH_2_–O–CH_2_–OCH_2_OH	86–87	1.34	0.56	-	0.45	2.20	1.01	-	0.63
HOCH_2_–O–CH_2_–OCH_2_OH	90–91	1.56	0.94	0.37	0.49	3.26	1.28	0.27	0.35
H(CH_2_O)_n_OCH_2_OCH_3_	94–95	0.11	-	-	0.17	0.20	-	0.79	-
	Total	6.72	4.17	2.67	1.16	12.32	6.17	3.79	6.85
–NH–CH_2_–O–CH_3_	73–74	1.97	1.87	0.57	4.74	2.20	1.01	0.65	2.25

M/E: the ratio of methylene bridges over ether bridges.
